# Focal-Plane Sensing-Processing: A Power-Efficient Approach for the Implementation of Privacy-Aware Networked Visual Sensors

**DOI:** 10.3390/s140815203

**Published:** 2014-08-19

**Authors:** Jorge Fernández-Berni, Ricardo Carmona-Galán, Rocío del Río, Richard Kleihorst, Wilfried Philips, Ángel Rodríguez-Vázquez

**Affiliations:** 1 Institute of Microelectronics of Seville (IMSE-CNM), CSIC-Univ. Sevilla, calle Américo Vespucio s/n, Seville 41092, Spain; E-Mails: rcarmona@imse-cnm.csic.es (R.C.-G.); rocio@imse-cnm.csic.es (R.R.); angel@imse-cnm.csic.es (Á.R.-V.); 2 Ghent University/iMinds/TELIN-IPI, St-Pietersnieuwstraat 41, Ghent B-9000, Belgium; E-Mails: rkleihor@telin.ugent.be (R.K.); philips@telin.ugent.be (W.P.)

**Keywords:** visual sensor networks, Internet of Things (IoT), privacy, security, vision sensor, focal-plane processing, obfuscation, pixelation, granular space, feature extraction

## Abstract

The capture, processing and distribution of visual information is one of the major challenges for the paradigm of the Internet of Things. Privacy emerges as a fundamental barrier to overcome. The idea of networked image sensors pervasively collecting data generates social rejection in the face of sensitive information being tampered by hackers or misused by legitimate users. Power consumption also constitutes a crucial aspect. Images contain a massive amount of data to be processed under strict timing requirements, demanding high-performance vision systems. In this paper, we describe a hardware-based strategy to concurrently address these two key issues. By conveying processing capabilities to the focal plane in addition to sensing, we can implement privacy protection measures just at the point where sensitive data are generated. Furthermore, such measures can be tailored for efficiently reducing the computational load of subsequent processing stages. As a proof of concept, a full-custom QVGA vision sensor chip is presented. It incorporates a mixed-signal focal-plane sensing-processing array providing programmable pixelation of multiple image regions in parallel. In addition to this functionality, the sensor exploits reconfigurability to implement other processing primitives, namely block-wise dynamic range adaptation, integral image computation and multi-resolution filtering. The proposed circuitry is also suitable to build a granular space, becoming the raw material for subsequent feature extraction and recognition of categorized objects.

## 1. Introduction

The Internet-of-Things (IoT) paradigm envisions an addressable continuum of interconnected smart objects exchanging information via the Internet [1]. One of the major objectives for this paradigm is minimum human intervention, targeting autonomous devices seamlessly integrated in our daily lives without explicit instructions [2]. In this context, sensor networks arise as an essential component of the IoT [3]. They take on the task of collecting data from the physical world, leading to the eventual activation of the actuators that carry out the decided actions. In terms of the measurements taken by the sensors, we can distinguish between scalar and non-scalar magnitudes. Scalar magnitudes, like temperature, pressure or humidity, have traditionally defined the application domains of sensor networks, e.g., precision agriculture [4], structural health [5] or meteorological analysis [6]. However, ongoing advances in electronics, sensor technologies, embedded hardware and software are enabling the incorporation of non-scalar magnitudes into the catalog of sensing capabilities of these networks. Specifically, the incorporation of vision means a challenge due to the strong restrictions imposed by the scarcity of resources—power, processing and memory—at the network nodes [7].

Before dealing with demanding low-level system requirements, a critical issue at the highest abstraction level must be carefully addressed first: privacy. Pervasive surveillance by networked image sensors creates legal and societal concerns. Citizens' sensitive information can be put at risk, causing people to reject the technology. Fortunately, identifiable personal data—faces, clothes, wearables *etc.*—are usually not needed to perform meaningful visual analysis in prospective application scenarios of visual sensor networks like retail analytics, factory monitoring or elderly care. Time spent by customers in front of a showcase, trajectories of workers around a manufacturing site or fall detection in a nursing home are three examples where video analytics can be realized without compromising privacy by discarding identifiable information early on. However, the concerns are understandably still there, because current smart imaging systems rely on a significant number of key software components to prevent leaking sensitive information. The implicitly trusted software base includes the operating system, the network stack, system libraries, *etc.* It is not possible to provide complete assurance about the potential security and privacy flaws contained in this software [8]. Even widely adopted cryptography libraries are not free of such flaws [9]. A possible approach to overcome these limitations is to convey protection as close to the sensing device as possible. In this regard, the system architecture presented in [10] constitutes state-of-the-art work related to privacy protection in camera networks. A strong separation between specific camera modules—hardware and software—accessing sensitive raw data and those ones devoted to application tasks is proposed. While this approach features a good tradeoff between security and system flexibility, there is still a number of trusted software components involved in the operation of the secure sensing unit. The ideal framework would be a front-end vision sensor delivering a data flow stripped of sensitive information. On-chip implementation of such stripping would still have to accommodate some degree of reconfigurability in order to balance protection and viability of the video analytics required by particular algorithms. Once protection measures are embedded on-chip at the front-end sensor of each network node, the number of trusted components as well as the impact of potential software flaws are significantly reduced.

Despite the importance of privacy for boosting the adoption of visual sensor networks, most prototype image sensors tailored for saving node resources do not address any protection measures in this respect [11–14]. Monolithic “light-in answer-out” system-on-a-chip solutions have been reported [15]. In this case, the sensor includes a small array of photo-diodes capturing 128 × 128-px images that feed an on-chip pipeline processing architecture. Images are never delivered off-chip. Extremely high performance is accomplished, but at the cost of low resolution and complex hardwired program control patterns. No alternative is considered either for applications requiring external storage of obfuscated images. Another privacy-aware approach for sensor networks is address-event representation (AER) [16]. Instead of full-resolution images, AER-based sensors constrain their outputs to codified events triggered by specific features like motion, light saturation or spatio-temporal contrast. The inherent filtering process of their operation removes sensitive data, but also most of the scene details, significantly reducing the scope of the video analytics applicable.

In this paper, we present a low-power vision sensor aiming at balancing processing flexibility and privacy protection. Sensitive image regions can be obfuscated through tunable pixelation at the focal plane. This tuning permits to preserve detailed image information if needed. The reconfiguration of the chip to dynamically accommodate vision algorithm requirements takes place on-the-fly. Other low-level processing primitives, like block-wise high dynamic range (HDR), integral image computation and multi-resolution filtering, can also be implemented by the prototype, boosting its scope of application. Plentiful experimental results are provided. Finally, the proposed sensing-processing scheme is also suitable to build a granular space [17] by exploiting focal-plane distributed memory. This granular space constitutes the raw material for subsequent recognition of categorized objects. It can also be adjusted, if afforded by the targeted application, to operate with coarse enough granule scales for the sake of privacy. In such a case, we would be concurrently combining purposeful power-efficient focal-plane processing and privacy protection.

## 2. Focal-Plane Sensing-Processing

**Figure 1.**
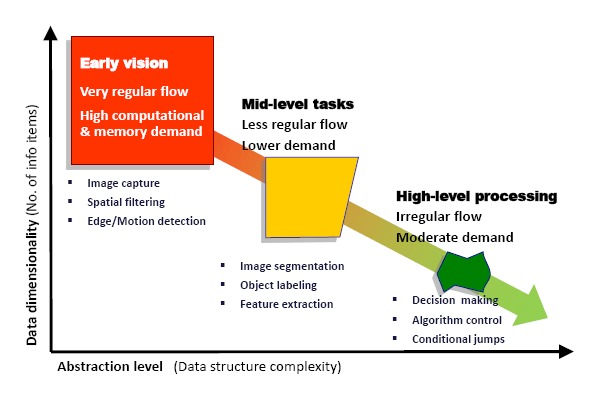
Comparison of data dimensionality *vs.* abstraction level for artificial vision. Early vision represents the critical stage in terms of computational and memory demands.

Artificial vision, understood as the process of automatically extracting meaningful information from visual stimulus, is usually divided into three consecutive stages: early vision, mid-level tasks and high-level processing [18]. If we compare these stages in terms of data dimensionality, *i.e.*, the number of information items *versus* the abstraction level, that is, the complexity of the data, a chart like that of Figure 1 can be depicted. Early vision operates on each and every pixel resulting from the raw readings of the sensor, but features a very regular computational flow: the same calculations are repeatedly carried out on every pixel. Mid-level tasks do not operate on raw data, but on a smaller amount of information of a higher abstraction level. Finally, high-level processing, of a cognitive nature, provides the semantic interpretation of the scene. It operates on a very reduced amount of data under a very irregular instruction flow. From this global picture of the vision process, we can conclude that early vision represents the critical stage in terms of computational and memory demands.

**Figure 2.**
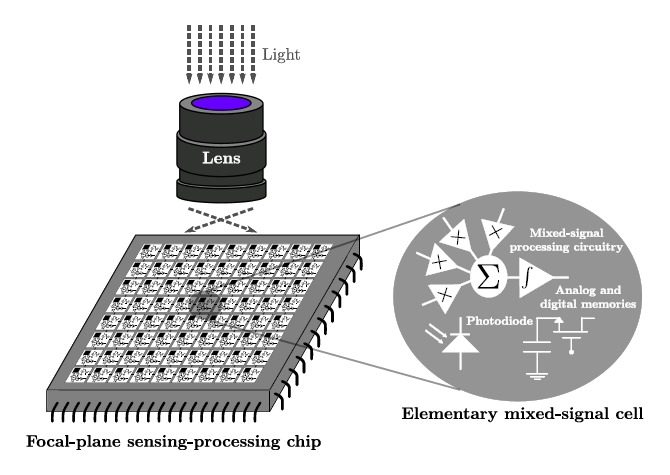
Sensing-processing vision chips capture and process images simultaneously at the focal plane by means of an array of interconnected elementary mixed-signal cells, also known as multi-functional pixels.

Focal-plane sensing-processing [19] is arguably the best architectural approach reported concerning adaptation to the particular characteristics of early vision. It is inspired in the operation of natural vision systems where a front-end device, the retina, does not only acquire, but also pre-processes the visual information. As a result of this pre-processing, the amount of data transmitted through the optic nerve to the brain gets compressed by a factor of ∼100 [20]. Different simplified representations of the scene being observed are rendered at this stage, speeding up its interpretation by the visual cortex. Focal-plane sensor-processor chips mimic this processing arrangement, as shown in a simplified way in Figure 2. An array of interconnected elementary mixed-signal cells, also known as multi-functional pixels, plays the role of front-end device in this case. The array usually operates in single-instruction multiple-data (SIMD) mode featuring concurrent processing and distributed memory. Focal-plane architectures can also benefit from incorporating analog circuitry just at the point where the analog data feeding the processing chain are sensed. These analog circuits can reach higher performance in terms of speed, area and power consumption than digital circuitry while exploiting the moderate accuracy requirements of early vision tasks. Numerous low-level image processing primitives have been successfully implemented following this scheme: convolution filtering [21,22], programmable blurring [23], spatial [24] and temporal [25] contrast extraction, background subtraction [26], image compression [27] or high dynamic range imaging [28], among others. Even academic [29] and commercial [30] general-purpose vision systems based on focal-plane processing have been reported.

## 3. A QVGA Focal-Plane Sensor-Processor Chip with Multi-Functional Pixels

### 3.1. Chip Architecture

The concept of focal-plane sensing-processing just described arises as the best framework to explore privacy protection for visual sensor networks. First of all, hackers will find it extremely difficult to tap sensitive information obfuscated from the very point where it is captured. Second, our ultimate objective is the implementation of a low-power high-performance vision system suitable for the scarce resources available at network nodes. In this regard, focal-plane sensing-processing features an architecture adapted for the efficient exploitation of the inherent properties of vision.

**Figure 3.**
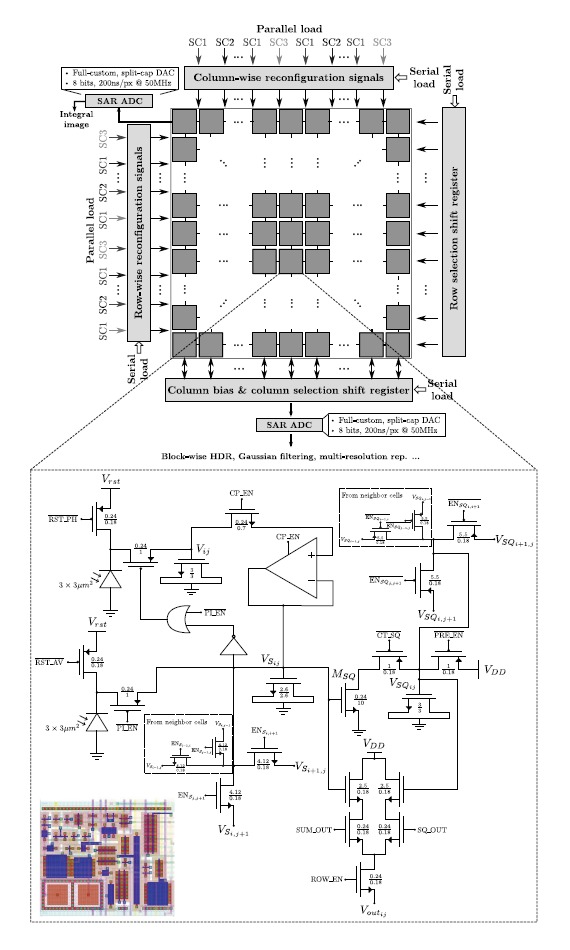
Architecture of the proposed focal-plane sensor-processor chip along with the schematic and layout of the multi-functional mixed-signal pixel.

All in all, the proposed prototype vision sensor presents the floor plan depicted in Figure 3. An array of four-connected mixed-signal multi-functional pixels constitutes its operative core. This array can be reconfigured block-wise by peripheral circuitry. The reconfiguration patterns are loaded serially into two shift registers that determine respectively which neighbor columns and rows can interact and which ones stay disconnected. There is also the possibility of loading in parallel up to six different patterns representing six successive image pixelation scales. This is achieved by means of control signals distributed regularly along the horizontal and vertical dimensions of the array [31]. The reconfiguration signals coming from the periphery map into the signals EN_*S*_*i,i*+1__, EN_*S*_*j,j*+1__, $\bar{{\text{EN}}_{SQ_{i,j+1}}}$ and $\bar{{\text{EN}}_{SQ_{j,j+1}}}$ at pixel level, where the coordinates (*i*,*j*) denote the location of the array cell considered. These signals control the activation of MOS switches for charge redistribution between the nMOS capacitors holding the voltages *V_S_ij__* and *V_SQ_ij__*, respectively. Charge redistribution is the primary processing task that supports all of the functionalities of the array, enabling a low-power operation. Concerning A-to-D conversion, there are four eight-bit SAR ADCs. These converters, based on a split-cap DAC, feature a tunable conversion range, including rail-to-rail, and a conversion time of 200 ns when clocked at 50 MHz. Two of them provide integral imaging. The other two convert the pixel voltage *V_out_ij__* corresponding to the selected output of the source followers associated with *V_S_ij__* and *V_Q_ij__*. The column and row selection circuitry is also implemented by peripheral shift registers where a single logic “1” is shifted according to the location of the pixel to be converted. The power consumption of the chip ranges from 42.6 mW for high dynamic range operation to 55.2 mW for integral image computation at 30 fps. It has been fabricated in a standard 0.18 μm CMOS process. The main characteristics of the chip are summarized in Table 1. A comparison with state-of-the-art focal-plane sensor-processor chips is presented in Table 2. Our prototype embeds the greatest functionality while featuring the largest resolution and the smallest pixel pitch, with a cost in terms of reduced fill factor.

**Figure 4.**
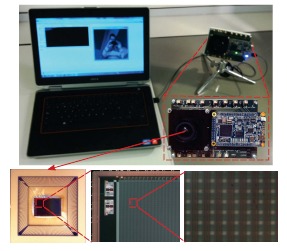
Snapshot of the test board while capturing images, a chip photo and two microphotographs showing, respectively, details of the upper left part of the chip and some multi-functional pixels.

For test purposes, the vision sensor has been embedded into a system based on the commercial FPGA-based *DE0-Nano* board from terasIC. The operation of the chip is fully controlled through 85 digital signals and 21 analog signals, thus enabling its integration with high-level power management schemes [35,36] in order to set low-power operation and idle states. The test system, together with some microphotographs of the chip, can be seen in Figure 4. The output data flow provided by the sensor is stored in the internal memory of the FPGA for its subsequent serial transmission to a PC through a USB interface. The data rearrangement and image visualization in the PC are implemented by making use of OpenCV functionalities.

### 3.2. On-Chip Early Vision

#### 3.2.1. Privacy Protection through Programmable Pixelation

Various techniques for privacy protection have been reported in the literature. The most basic form is blanking [37], where sensitive regions are completely removed from the captured images. No behavioral analysis is possible in this case; only the presence and location of a person can be monitored. Other alternatives that do enable such analysis are obfuscation and scrambling [38]. Concerning obfuscation, pixelation of sensitive regions provides the best performance in terms of balance between privacy protection and intelligibility of the surveyed scene when compared to blurring and masking filters [39,40]. New techniques for obfuscation, like warping [41] or cartooning [42], have been recently proposed.

**Figure 5.**
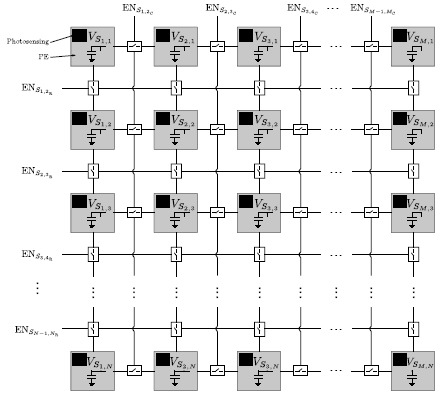
Simplified scheme of how the charge redistribution can be reconfigured column-wise and row-wise from the periphery of the proposed focal-plane array.

The on-chip programmable pixelation is achieved by combining focal-plane reconfigurability, charge redistribution and distributed memory. After photointegration, the corresponding pixel values are represented by the voltages *V_ij_* distributed across the array. These pixel values can be copied in parallel into the voltages *V_S_ij__* by enabling the analog buffer included at each elementary cell. This copy process takes about 150 ns for the whole array. It is not destructive with respect to the original voltages *V_ij_*, which is crucial to accomplish focal-plane obfuscation without artifacts, as explained below. Once the voltages *V_S_ij__* are set, the next step consists in establishing the adequate interconnection patterns according to the image regions to be pixelated and the required degree of obfuscation. These patterns, when activated by the corresponding control signal, will enable charge redistribution among the connected capacitors holding *V_S_ij__*, *i.e.*, the image copy. Figure 5 shows a simplified scheme of how the charge redistribution can be reconfigured column-wise and row-wise from the periphery of the proposed focal-plane array. Every pair of neighboring columns and rows shares a common interconnection signal that is independent of the interconnection signals for any other pair. When the interconnections of consecutive columns and rows are enabled, voltage averaging, *i.e.*, charge redistribution, will take place within the resulting block. Otherwise, the pixel values remain unchanged.

**Figure 6.**
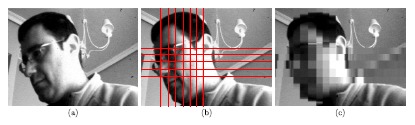
(**a**) Original image captured by the chip. (**b**) Patterns for pixel interconnection. (**c**) Undesired pixelation artifacts out of the region of interest distorting the image content.

**Figure 7.**
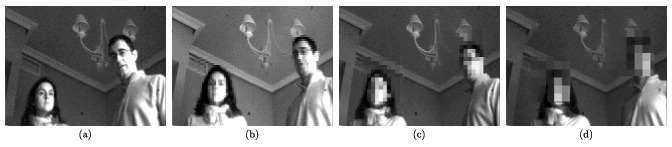
On-chip obfuscation without artifacts by exploiting the A-to-D conversion stage: (**a**) original image captured by the chip; (**b**) pixelation with 4 × 4-px blocks; (**c**) pixelation with 8 × 8-px blocks; (**d**) pixelation with 16 × 16-px blocks.

An example of the focal-plane obfuscation attainable by applying this operation is shown in Figure 6. The first snapshot represents an image captured by the chip. The interconnection patterns established *ad hoc* for the pixelation of the face in the scene are highlighted in the second picture. Finally, the third image depicts the resulting focal-plane representation. It can be seen that the existence of a single control signal for the interconnection of all of the cells along particular neighbor columns and rows generates spurious blocks within which partial charge redistribution also occurs. The consequent artifacts significantly reduce the amount of useful information contained in the image, distorting its content. However, we can overcome this problem by exploiting the distributed memory inherent to the sensing-processing array. Bear in mind that the image in Figure 6c is the outcome after photointegration, pixel copy, interconnection setting, charge redistribution and A-to-D conversion. This last stage is key to remove the aforementioned artifacts. During A-to-D conversion, we copy the corresponding original pixel value still stored in the capacitors holding *V_ij_* for the regions where full resolution is affordable in terms of privacy. Otherwise, averaging is allowed. On-chip obfuscation without artifacts can thus be achieved, as shown in Figure 7. In this case, we set interconnection patterns for progressively coarser simultaneous pixelation of two different image regions containing faces.

**Figure 8.**
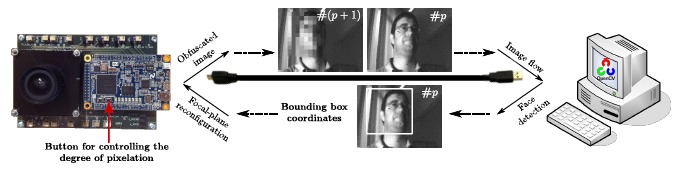
Real-time focal-plane reconfiguration conducted by the Viola-Jones frontal face detector. If faces are detected, the coordinates of the corresponding bounding rectangles are sent back to the test board for the vision sensor to reconfigure the image capture on-the-fly.

Finally, as an example of on-the-fly focal-plane reconfiguration conducted by a vision algorithm, we set up a closed loop between our test system and the Viola-Jones frontal face detector provided by OpenCV, as depicted in Figure 8. The sensor captures images that are sent to a PC from the test board. The Viola-Jones detector is run on these images on the PC. If faces are detected, the coordinates of the corresponding bounding rectangles are sent back to the test board for the vision sensor to reconfigure the image capture in real time. Pixelation of the face regions will take place from that moment on at the focal plane. The degree of pixelation of these regions is adjustable through a button of the test board. A sequence extracted during the execution of this loop can be download from [43]. Notice that all of the reconfiguration and control of the array must be carried out externally for this prototype. The FPGA of the DE0-Nano board plays such a role in the test system.

#### 3.2.2. Block-Wise HDR

One of the fundamental problems that visual sensors have to cope with is the inspection of scenes featuring a wide range of illuminations. In such cases, assuming the usual photocurrent integration mode, the application of the same exposure time across the pixel array leads to either over-exposed or under-exposed regions. The consequent low contrast and missing details can make the processing of these regions unfeasible. The processing primitive described next is specially suitable for vision algorithms requiring tracking of regions of interest (ROI) [44,45] in such circumstances.

**Figure 9.**
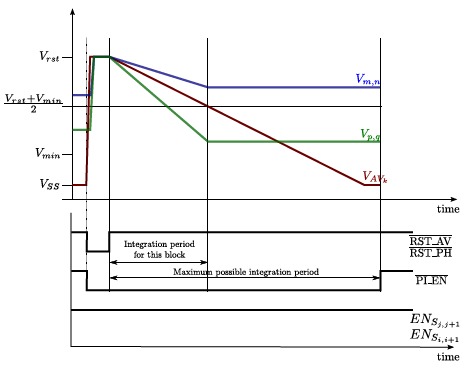
Timing diagram illustrating the operation of block-wise HDR. The dynamics of two pixels belonging to the same block along with the corresponding local averaging voltage is depicted.

The two photodiodes and two sensing capacitances included at the elementary cell in Figure 3 are required to implement this low-level operation. Their reset to the voltage *V_rst_*, which coincides with the upper limit of the signal range, is controlled through the signals $\bar{\text{RST\_PH}}$, $\bar{\text{RST\_AV}}$ and $\bar{\text{PI\_EN}}$. When the three of them are set to logic “0”, the reset of the so-called pixel photodiode, averaging photodiode and averaging capacitance is immediately enabled. If the voltage *V_S_ij__* starts from a value above the input threshold voltage of the inverter, *V_th_inv__*, the reset of the pixel capacitance holding *V_ij_* is concurrently initiated. Otherwise, as is the case in the illustrating timing diagram of Figure 9, this reset will be delayed slightly until *V_S_ij__* reaches that threshold. A key aspect of the circuit is that the inverter must be designed in such a way that *V_th_inv__* is located just at the middle point of the signal range, that is:

(1) $$V_{{\text{th}}_{\text{inv}}}=\frac{V_{\text{rst}}+V_{\text{min}}}{2}$$

where *V_min_* is the lower limit of the signal range. Thus, the integration interval for each block will be determined asynchronously according to its incident illumination, once $\bar{\text{RST\_PH}}$ and $\bar{\text{RST\_AV}}$ are set back to “1”. At that time instant, photointegration begins concurrently in both the pixel capacitance and the averaging capacitance. However, while in the former it is carried out in an isolated way, charge redistribution takes place in the latter among the averaging capacitances interconnected through the switches controlled by EN_*S*_*i,i*+1__ and EN_*S*_*j,j*+1__. Keep in mind that these signals come from the peripheral circuitry previously mentioned. As a result, the voltage excursion (For the sake of simplicity, we define the voltage excursion as the difference between the initial voltage, *V_rst_,* and the final voltage. This permits getting rid of the minus sign.) due to photointegration for each pixel within a certain block *k* is given by:

(2) $$\Delta V_{\text{ij}}=\frac{I_{{\text{ph}}_{\text{ij}}}}{C}T_{k}$$

where *I_ph_ij__* denotes the sum of the photogenerated current plus the dark current and *T_k_* is the photointegration period, which is the same for all of the pixels composing the block. It must be observed that, since the averaging capacitances are interconnected, the following equation holds:

(3) $$\sum_{\forall i,j\in k}I_{{\text{ph}}_{\text{ij}}}=-\text{W HC}\frac{{\text{dV}}_{{\text{AV}}_{k}}}{\text{dt}}$$

where *W* × *H* are the dimensions of the block, in pixels, and *V_AVk_* is the voltage at the averaging capacitances holding *VSij*, the same at each of them due to the charge redistribution taking place constantly. The photointegration at the pixel capacitances will finish when *V_AV_k__* reaches *V_th_inv__*, that is, from Equation (3):

(4) $$T_{k}=\text{W HC}\frac{V_{\text{rst}}-V_{{\text{th}}_{\text{inv}}}}{{\text{∑}}_{\forall i,j\in k}I_{{\text{ph}}_{\text{ij}}}}$$

which can be expressed, taking into account Equation (1), as:

(5) $$T_{k}=\frac{C}{2}\frac{\Delta V_{{\text{ij}}_{\text{MAX}}}}{{Ī}_{{\text{ph}}_{k}}}$$

where Δ*V_ij_MAX__* = *V_rst_* − *V_min_* represents the maximum pixel excursion and *Ī_ph_k__* is the block average current generated during the photointegration period. If the effect of the dark currents can be neglected, this current is directly proportional to the average incident illumination on the block. By substituting Equation (5) in Equation (2), the following voltage excursion for each pixel is obtained:

(6) $$\Delta V_{\text{ij}}=\frac{\Delta V_{{\text{ij}}_{\text{MAX}}}}{2}\frac{I_{{\text{ph}}_{\text{ij}}}}{{Ī}_{{\text{ph}}_{k}}}$$

where we can see that the maximum pixel illumination to be detected without saturation is double the average illumination of the block. This property, when applied to the whole pixel array, means a DR enhancement of only 6 dB [46]. However, when confined to any particular rectangular-shaped block, it endows our hardware with the capability of dealing with regions of interest (ROIs) across frames featuring a total intra-scene dynamic range of up to 102 dB. The dynamics of two arbitrary pixels belonging to the same block along with the corresponding local averaging voltage is depicted in Figure 9. *V_m,n_* represents a pixel whose illumination is below the average block illumination, whereas *V_p,q_* represents a pixel receiving illumination above that average. Notice that the integration period for all of the pixels of the block, including *V_m,n_* and *V_p,q_* arbitrarily selected for representation, ends at the same time instant without requiring any external control. That instant is set when the sensing of the average incident illumination, denoted by *V_AV_k__*, reaches the middle point of the signal range. Nevertheless, the signal $\bar{\text{PI\_EN}}$ permits one to finish the photointegration for those regions whose illumination is so low that it does not reach that middle point within the limit established by the prescribed frame rate. This example can be extended to the whole focal-plane array. Thus, once the different blocks for the next image to be captured are established, a maximum photo-integration period for all of them starts to run. During this period, each block adjusts automatically and independently its integration time according to its particular mean illumination.

**Figure 10.**
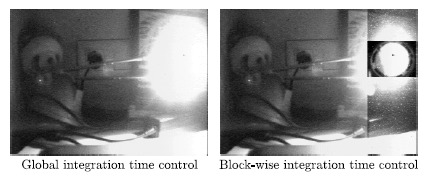
On-chip block-wise intra-frame integration time control. Global control of the integration time is applied to the left image. In the right image, the integration time of the region around the center of the lamp adjusts locally and asynchronously to its mean illumination.

An example of this operation is shown in Figure 10. Global control of the integration time is applied to the left image. All pixels undergo the same integration time, which is set to 500 ms according to the mean illumination of the scene. Details about the lamp are missed due to the extreme deviation with respect to this mean illumination. However, such details can be retrieved by confining the control of the integration period to the region of interest, as can be seen in the right image. In this case, the integration time of the region around the center of the lamp adjusts locally and asynchronously to its mean illumination, stopping the photointegration at around 400 μs in that particular area while it continues at the remaining regions.

#### 3.2.3. Integral Image

The so-called integral image is a common intermediate image representation used by well-known vision algorithms, e.g., the Viola–Jones framework for object detection [47]. It is defined as:

(7) $$II(x,y)=\overset{x}{\underset{x^{\prime }=1}{\text{∑}}}\overset{y}{\underset{y^{\prime }=1}{\text{∑}}}I(x^{\prime },y^{\prime })$$

where *I*(*x*, *y*) represents the *M* × *N*-px input image with *x* ∈ [1, *M*] and *y* ∈ [1, *N*]. That is, each pixel composing *II*(*x*, *y*) is given by the sum of all the pixels above and to the left of the corresponding pixel at the input image. We exploit the focal-plane reconfigurability sketched in Figure 5 to compute the integral image. For example, the computation of its first row simply requires to disable all row connections between pixels and then progressively enable column connections, and thereby averaging, for each pixel of the row. This control pattern repeats for the rest of the rows provided that the interconnection between them is progressively enabled as the computation of the integral image progresses. In order to deal with the extremely wide signal range required to represent an integral image, charge redistribution plays a key role as the underlying physical operation supporting the computation at the focal plane. It permits to keep the signal swing within the range of individual pixels, no matter how many pixels of the original image are involved in the computation of the current integral image's pixel. The average value obtained for each case simply requires externally keeping track of the position of the pixel being calculated. Thus, we only need to multiply that average value by the number of row and column associated with the corresponding pixel. In other words, the array is capable of computing an averaged version of the integral image mathematically described as:

(8) $$II_{av}(x,y)=\frac{1}{x\cdot y}\overset{x}{\underset{x^{\prime }=1}{\text{∑}}}\overset{y}{\underset{y^{\prime }=1}{\text{∑}}}I(x^{\prime },y^{\prime })$$

**Figure 11.**
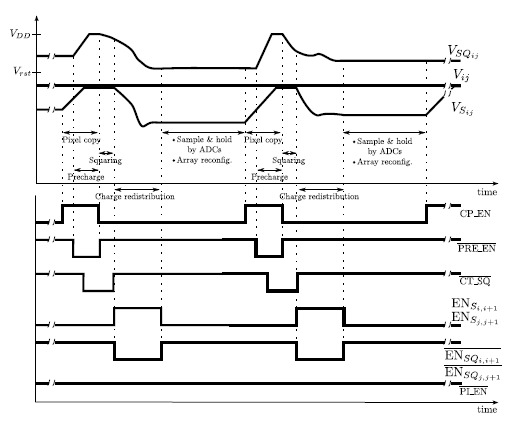
Timing diagram showing the signals involved in the computation of two consecutive integral image pixels.

The array can also compute an averaged version of the square integral image. To this end, we make use of the squarer experimentally tested and reported in [48]. Thus, by precharging the capacitor holding *V_SQ_ij__* to *V_DD_* and exploiting its discharge for a short period of time through the transistor *M_SQ_* working in the saturation region, the value of the pixel square can be computed. Then, charge redistribution takes over, just as for the integral image. Indeed, apart from the required previous computation of the pixel square, the procedure to obtain the averaged versions of the integral image and the square integral image is exactly the same, applied respectively to the voltages *V_S_ij__* and *V_SQ_ij__* at the pixel level. A timing diagram showing two consecutive computations of integral image pixels is depicted in Figure 11. In order to read out and convert these pixels, we must simply connect *V*_*S*_1,1__ and *V*_*SQ*_1,1__ to the respective analog-to-digital converters. These voltages will always contain the targeted calculation for each pixel, according to the definition of integral image and the proposed hardware implementation based on charge redistribution.

**Figure 12.**
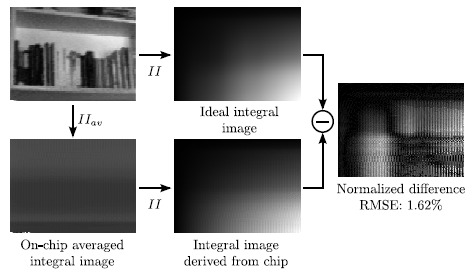
On-chip focal-plane integral image computation and comparison with the corresponding off-chip ideal computation.

An example of on-chip integral image computation is shown in Figure 12. In this case, we can visualize the averaged integral image delivered by the chip and the integral image that can be directly derived from it. This integral image is compared to the ideal integral image obtained off-chip with MATLAB from the original image captured by the sensor, attaining an RMSE of 1.62%.

#### 3.2.4. Multi-Resolution Filtering

**Figure 13.**
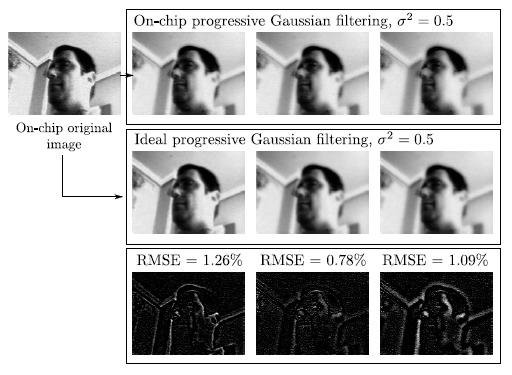
On-chip focal-plane Gaussian filtering and comparison with the corresponding off-chip ideal computation.

The combination of focal-plane reconfigurability, charge redistribution and distributed memory enables subsequent reduced kernel filtering by adjusting which pixels merge their values and in which order. Very useful image filtering kernels, like the binomial filter mask for image Gaussian smoothing or the Sobel operators for edge detection, fall into the category of reducible kernels [49]. Operating on the pre-processed image with one of them represents a smaller number of operations per pixel than realizing all of the multiply-accumulate operations needed to apply the corresponding original kernels. Memory accesses are reduced in the same fraction. An example of Gaussian filtering is depicted in Figure 13.

## 4. Guidelines for Future Work: Feature Extraction from On-Chip Granular Space

This section describes how the proposed on-chip reconfiguration scheme can be adapted to speed up object recognition and even to concurrently combine this task with privacy protection. Note that our sensor was not designed with this application in mind. The connection between the different concepts involved came later when exploring image features suitable for our focal-plane sensing-processing approach. Therefore, key parameters of the prototype have not been tuned for reaching real-time operation in this case. Nevertheless, we consider that the hardware-software co-design framework sketched next is a completely new idea in the field of embedded vision systems. It will constitute a solid base supporting the process of setting specifications for a future sensor chip.

### 4.1. Granular Space

There are two major approaches for generic object recognition in computer vision: window-based object representations and part-based object representations [50]. Window-based representations perform better for recognition of rigid objects with a certain regularity in their spatial layout. They implement a holistic description of appearance based on a dense sampling of features obtained at multiple scales. To determine the presence or absence of a certain object in a new image, this approach makes use of a sliding window testing all possible sub-windows of the image with a previously trained classifier. On the other hand, part-based representations usually perform better for non-rigid deformable objects, since these describe the appearance of an object through local parts following a certain geometric interconnection pattern. They therefore incorporate more detailed spatial relations into the recognition procedure, requiring complex search procedures for the detection of image keypoints. Conversely, they require fewer training examples. Generally speaking, it has been observed that, given sufficient training data, the discriminative sliding-window models outperform when it comes to recognizing typical targeted objects for embedded vision like faces or front/back views of pedestrians.

**Figure 14.**
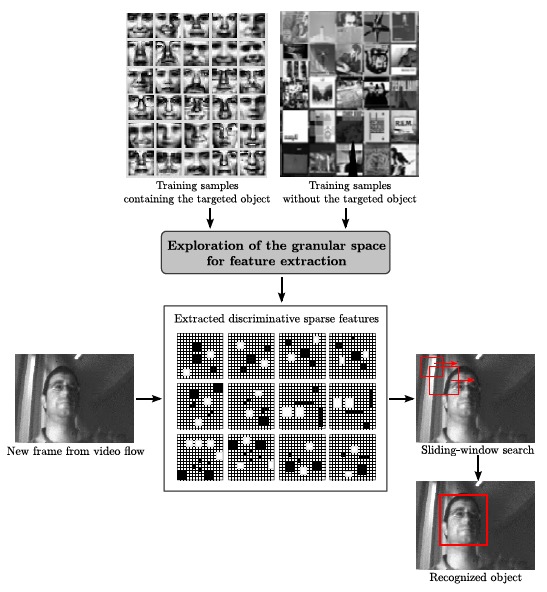
Object recognition pipeline based on the granular space. The white and black granules are respectively weighted positively and negatively when it comes to attaining a representative value of the corresponding sparse feature.

The so-called granular space for feature extraction belongs to the sliding-window category. It was first defined in [17], being applied for multi-view face detection. It simply consists in a multi-resolution space composed of the so-called granules that observe each image instance at different locations and scales. The granules are represented by the average value of their constituting pixels. By combining different granules across the image, a new sparse feature is obtained. A large number of these sparse features are learned during training for a particular object. They are later analyzed during real operation for new images where such an object could be present. A scheme of the procedure is depicted in Figure 14. The white and black granules are respectively weighted positively and negatively when it comes to attaining a representative value of the corresponding sparse feature. This value is then compared with that of another feature or with a threshold, according to the classification technique applied.

The original work in [17] has been extended during the last few years mostly by introducing variations in the training and feature search strategy [51–54]. The recognition of other objects, like pedestrians, has also been addressed. Still, the raw material feeding the whole processing chain keeps being the granular space. It is on building this space where focal-plane sensing-processing can contribute to boosting the global performance.

### 4.2. On-Chip Processing

**Figure 15.**
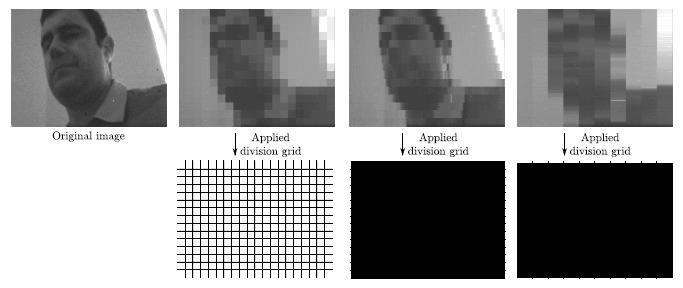
Examples of focal-plane division grids concurrently providing granules of different sizes across the whole image. The resulting distortion can be exploited for privacy protection as long as the subsequent classifier can afford to work with granules of a large enough minimum size.

The granular space is ultimately based on block-wise averaging across the image plane. Coincidentally, the operation of averaging, physically supported by charge redistribution, is one of the most efficient computations to be realized in parallel at the focal plane. As we described in Section 3.2.1, we have successfully implemented a focal-plane reconfiguration scheme for block-wise programmable averaging in our prototype vision chip. This scheme permits to apply division grids enabling the concurrent computation of rectangular-shaped granules of any size across the whole image, as depicted in Figure 15. For the grids of this example, the focal-plane array required only ∼200 ns to respectively calculate 300, 2400 and 1220 granules in parallel. Unfortunately, the bottleneck for real-time operation arises during the A/D conversion. As mentioned earlier, the chip was not designed to provide several scales of the granular space per frame in real time as would be required by an algorithm of object recognition based on sparse feature extraction in such a space. Specifically, we should have included additional in-pixel memory and additional A/D converters working in parallel according to the targeted frame rate and number of scales. Finally, notice that the computation of granules at the focal plane, while meaningful from the perspective of object recognition, implies a significant distortion of the image content. Such a distortion can be exploited for privacy protection as long as the subsequent classifier can afford to work with granules of a large enough minimum size.

### 4.3. Hardware-Software Co-Design Framework

A great deal of tradeoffs affecting both hardware and software will have to be considered for the design and implementation of a future network visual node based on the focal-plane generation of the granular space. Key parameters for the performance of such a node, like the resolution, the frame rate or the detection rate, must be determined by carefully adjusting design variables, like the pixel size, sensitivity or conversion time (hardware level), together with others, like the size of the granules or the training technique (software level). From our point of view, software simulations must first set the initial tentative specifications for the hardware. To this end, we are starting to build a tunable classifier based on features extracted from the granular space. The design loop will be closed by hardware simulations derived from the initial specifications provided by the classifier. These simulations will then feedback software simulations and *vice versa* in a constant process of parameter adjustment and performance evaluation. Eventually, we will obtain accurate requirements for a smart imager tailored for a privacy-aware vision system providing reliable object recognition at low power consumption.

## 5. Conclusions

The deployment of ubiquitous networked visual sensors seamlessly integrated in the Internet of Things is still a challenge to be accomplished. Privacy and power consumption are two major barriers to overcome. Once visual information is disconnected from private data and locally processed by low-power trusted computing devices, a wide range of innovative applications and services will be able to be addressed; applications and services now impossible to be implemented because current technology collides with legal constraints and public rejection. The ultimate objective is that such vision-enabled devices represent a concern not higher than lighting or door control based on automatic presence detectors. In this paper, we have reported our contribution to advance the state of the art regarding privacy and ultra-low-power in-node processing for visual sensor networks. A full-custom prototype vision sensor has been presented. Its most distinctive feature, in addition to power efficiency, is the reconfigurability of its focal-plane sensing-processing array. This reconfigurability enables real-time adaptation to specific requirements from vision algorithms. Particularly, we have demonstrated that concurrent obfuscation of different image regions can be achieved directly at the focal-plane. A new framework for merging focal-plane privacy protection and purposeful parallel processing has also been sketched. This framework, based on the extraction of sparse features from the granular space, will be our research focus in the short/mid-term.

## Figures and Tables

**Table 1.** Summary of the main chip characteristics.

**Technology**	0.18 μm 1.8 V 1P6M CMOS process
**Die Size (with Pads)**	7.5 mm × 5 mm
**Pixel Size**	19.59 μm × 17 μm
**Fill Factor**	5.4%
**Photodiode Type**	n-well/p-substrate
**Power Supply**	3.3 V (pads), 1.8 V (core)
**DSNU**	1.7%
**PRNU (50% Signal Range)**	3.5%
**ADC throughput**	5 MSa/s (200 ns/Sa)

**Power Consumption at 30 fps**	
Image capture	42.6 mW
Programmable pixelation	43.8 mW
Block-wise HDR	42.6 mW
Integral imaging	55.2 mW
Gaussian filtering	43.1 mW

**Table 2.** Comparison of the proposed prototype with state-of-the-art focal-plane sensor-processor chips.

**Reference**	[32]	[33]	[34]	This work
**Function**	Edge filtering, tracking, HDR	Gaussian filtering	2D optic flow estimation	HDR, integral image, Gaussian filtering, programmable pixelation
**Tech.** (μm)	0.18	0.18	0.18	0.18
**Supply** (V)	0.5	1.8	3.3	1.8
**Resolution**	64 × 64	176 × 120	64 × 64	320 × 240
**Pixel pitch** (μm)	20	44	28.8	19.6
**Fill factor** (%)	32.4	10.25	18.32	5.4
**Dynamic range** (dB)	105	-	-	102
**Power consumption** (nW/px·frame)	1.25	26.5	0.89	23.9
